# A Meta-Analysis of Statin Use and Risk of Hepatocellular Carcinoma

**DOI:** 10.1155/2022/5389044

**Published:** 2022-03-20

**Authors:** Yikai Wang, Wenjun Wang, Muqi Wang, Juanjuan Shi, Xiaoli Jia, Shuangsuo Dang

**Affiliations:** Department of Infectious Diseases, The Second Affiliated Hospital of Xi'an Jiaotong University, Xi'an, Shaanxi 710004, China

## Abstract

**Background:**

The use of statins is a potential protective factor against the development of hepatocellular carcinoma. Therefore, we conducted a meta-analysis to evaluate the contribution of statins to the risk of hepatocellular carcinoma.

**Methods:**

We searched for PubMed and EMBASE through January 2021.

**Results:**

Thirty-two studies (eighteen cohort, eleven case-control, and three randomized controlled trials) reporting 56,838 cases of hepatocellular carcinoma in 4,963,518 persons were included. Statin users were less likely to develop hepatocellular carcinoma than nonusers (adjusted odds ratio, 0.58; 95% CI: 0.51–0.67). Stratified analysis showed that statins reduced the risk of hepatocellular carcinoma in Asian and Western populations (odds ratio, 0.54 vs. 0.60). Besides, statins have protective effects against hepatocellular carcinoma after hepatitis B virus (odds ratio, 0.44; 95% CI: 0.22–0.85) and hepatitis C virus infections (odds ratio, 0.53; 95% CI: 0.49–0.57). Statins have protective effects on people with chronic liver disease (odds ratio, 0.52; 95% CI: 0.40–0.68) and on the general population (odds ratio, 0.60; 95% CI: 0.50–0.72). Lipophilic statins can prevent hepatocellular carcinoma (odds ratio, 0.51, 95% CI: 0.46–0.57), while hydrophilic statins cannot (odds ratio, 0.77, 95% CI: 0.58–1.02). The single-drug analyses showed that simvastatin (odds ratio, 0.53, 95% CI: 0.48–0.59), atorvastatin (odds ratio, 0.54, 95% CI: 0.45–0.64), rosuvastatin (odds ratio, 0.55, 95% CI: 0.37–0.83), lovastatin (odds ratio, 0.30, 95% CI: 0.15–0.62), and pitavastatin (odds ratio, 0.36, 95% CI: 0.17–0.75) had significant benefits. Further studies have shown that those in the high-dose group experienced better effects in preventing hepatocellular carcinoma (adjusted hazard ratio, 0.38 vs. 0.55). Further research found that the combined use of aspirin did not increase the chemoprevention effect of liver cancer (odds ratio, 0.57; 95% CI: 0.40–0.81). In addition, the preventive effect of statins improved with the extension of follow-up time (odds ratio, 0.54 vs. 0.65).

**Conclusion:**

Our meta-analysis shows that the use of statins is associated with a lower risk of liver cancer.

## 1. Introduction

Liver cancer is one of the most common cancers in the world, imposing substantial burdens on patients and society [1, 2]. Traditional treatment methods such as surgical resection, liver transplantation, and chemotherapy also have a good curative effect in the early stage of cancer. With the emergence of targeted drugs such as sorafenib, a new approach has been brought to the treatment of liver cancer. In recent years, immunotherapy has gradually emerged, especially the use of inhibitors targeting immune checkpoints, PD1, PDL1, and CTLA4, showing a good therapeutic prospect. However, when 70% of liver cancer patients go to a doctor, the disease has progressed to the middle and late stages [3], so it is still particularly important to prevent the occurrence and progression of liver cancer. However, there is still no preventive drug that can reduce the risk of liver cancer. Statins are mostly used to prevent and control cardiovascular diseases [4] and reduce plasma cholesterol levels [5]. In addition, increasing attention has been given to statins because of their anticancer effects [6, 7] on prostate cancer [8], colorectal cancer [9], and hepatocellular carcinoma (HCC) [10–12]. Previous investigations have shown that statins have the potential chemopreventive effects on liver cancer [13] in vivo and in vitro, mainly by inhibiting angiogenesis, tumor growth, and metastasis and inducing apoptosis [14–16]. However, some studies did not show any beneficial effects [17].

Accordingly, our group conducted this meta-analysis to evaluate the relationship between statin use and the risk of liver cancer; this updated meta-analysis has the benefits of including recently published studies and more subgroup analyses.

## 2. Methods

### 2.1. Subjects

The meta-analysis is reported based on the Meta-analysis Of Observational Studies in Epidemiology (MOOSE) checklist [18], following the Preferred Reporting Items for Systematic Review and Meta-Analysis (PRISMA) guidelines (ID: CRD42021268397). No ethics issues were involved in this research because our data were obtained from published studies.

### 2.2. Included Index Classifications

Randomized controlled trials (RCT), cohort studies, nested case-control studies, and case-control studies were included in this meta-analysis. The patients included were adults (18 years or older) who were ultimately diagnosed with liver cancer (diagnosed using any recognized diagnostic criteria), regardless of sex or race. In addition, the interventions were defined as any dose, route of administration, and duration of statin therapy (including fluvastatin, pravastatin, rosuvastatin, simvastatin, lovastatin, pitavastatin, atorvastatin, and cerivastatin). The control group could be a placebo or a nonstatin therapy.

Studies were excluded if they were animal experiments, reviews, meta-analyses, editorials, and published letters.

### 2.3. Treatment of Results

The risk of HCC in statin users was assessed first. We use the odds ratio (OR) to evaluate the risk of HCC. When the OR was less than 1, the use of statins had a protective effect against the occurrence of HCC. To explain the potential heterogeneity, experimental design, experimental location, etiology, presence of chronic liver disease, type of statins, and dosage of statins were investigated.

### 2.4. Search Strategy

**Figure 1:**
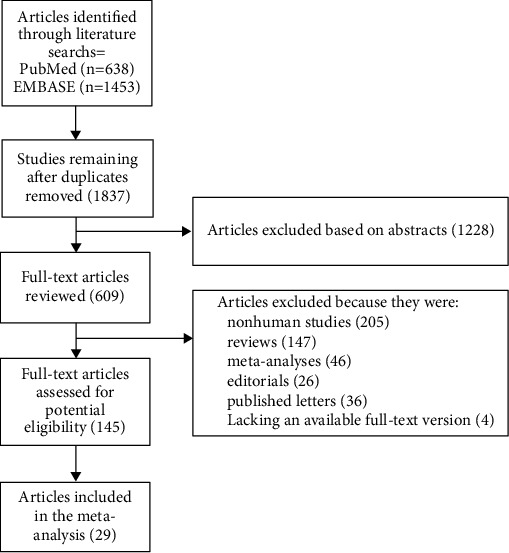
Flow diagram summarizing study identification and selection.

Two electronic databases containing articles published in English, namely, PubMed and EMBASE, were thoroughly searched to identify the studies that could be included in the analysis. Based on the guidance in the Cochrane handbook, we developed detailed strategies, including “statin” [MeSH term] or “statin” or “atorvastatin” or “fluvastatin” or “cerivastatin” or “lovastatin” or “pravastatin” or “rosuvastatin” or “simvastatin” or “pitavastatin” or “HMG-CoA reductase inhibitors” and “hepatocellular carcinoma” [MeSH terms] or “liver neoplasms” [MeSH term] or “liver cancer” or “HCC.” The reference lists of the studies included and related reviews were manually checked to identify other citations. To ensure that the qualified studies were correctly identified, two researchers independently read each report, and all the researchers resolved their differences by reaching consensus. The flowchart summarizing the study selection process is shown in Figure 1.

### 2.5. Data Extraction and Quality Evaluation

Data were extracted from all eligible studies by 2 independent researchers (Yikai Wang and Wenjun Wang). The following data were collected from each study: study design, country of origin of the study population, time period of the study, number of people included, outcome events, and adjusted confounding factors.

Data on the following confounding HCC risk factors were extracted from each study: age, sex, race, socioeconomic status, body mass index (BMI), diabetes (DM), the presence of cirrhosis, hepatitis B virus (HBV) infection, hepatitis C virus (HCV) infection, presence of alcoholic liver disease, and the use of angiotensin-converting enzyme inhibitors/nonsteroidal anti-inflammatory drugs/aspirin/metformin/proton pump inhibitors (PPIs)/nonstatin lipid-lowering drugs and lifestyle habits (Tables 1 and 2).

**Figure 2:**
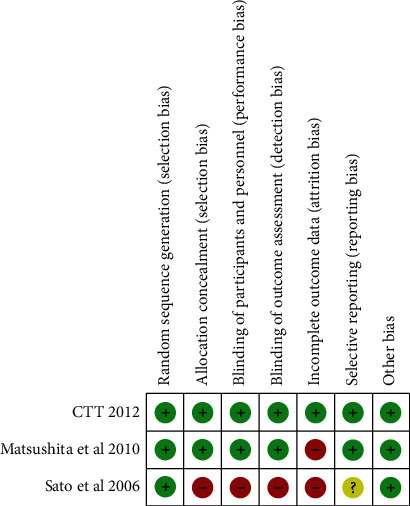
Quality of the RCT articles.

We used the Cochrane intervention system evaluation manual V.5.1.0 to evaluate the quality of RCTs. The risk of bias tool covers seven aspects: random sequence generation, allocation concealment, blinding of participants and personnel, blinding of outcome assessment, incomplete outcome data, selective reporting, and other bias. In the end, we classified each RCT into one of three levels: high risk, low risk, and unclear risk of bias. The results of the risk assessment are shown in Figure 2.

The quality of observational research was assessed using the Newcastle–Ottawa scale, which is one of the most commonly used rating systems for evaluating the quality of observational research in meta-analyses [19]. The scale is based on a star allocation system, in which up to nine stars are allocated based on the risk of bias in the three covered areas. Each study is scored according to the following three items: patient selection (four stars), comparability of study groups (two stars), and results/exposure assessment (three stars). The star rating system can be used to indicate the quality of each study. Fewer than seven stars indicate low quality, and more than seven stars indicate high quality. Two reviewers independently evaluated the risk of bias in the included studies. If there were any differences in their evaluations, a consensus was reached through group discussion and negotiation. If we had unresolvable questions, we directly asked the author.

### 2.6. Statistical Analysis

We considered *P* < 0.05 to indicate statistical significance, and all statistical tests were two-sided. For statistical analysis, we chose the commonly used software Review Manager (RevMan v5.4, Copenhagen). To obtain a more generalizable result, we used the random-effects model for the meta-analysis of the OR data because all potential heterogeneities were included in the construction of model [20]. By analyzing the adjusted OR (for case-control studies) in this study, the confounding variables were accounted for to a certain extent.

We used the *I*^2^ and Cochran's *Q* tests to evaluate heterogeneity, as these are often used to measure inconsistency among different studies. If the *I*^2^ value was >50% or *P* < 0.10, the heterogeneity was considered significant [21]. We used STATA 16.0 to draw a funnel plot and perform an Egger's regression asymmetry test.

## 3. Results

### 3.1. Search Results

We selected 638 and 1,453 articles from PubMed and EMBASE, respectively. After applying the inclusion criteria and deleting duplicate studies, 30 studies were finally selected for inclusion in the meta-analysis [17, 22–49]. In a total of 4,963,518 people, these studies reported 56,838 cases of HCC. The RCTs selected for the analysis included two prospective controlled trials of atherosclerotic heart disease, and these patient data were included in the analysis [47, 48]. Among the included studies, the cohort study conducted by Friedman et al. [33] gave the OR estimates for men and women separately, so we treated these datasets as two independent studies in the analysis.

### 3.2. Characteristics of Included Studies

The characteristics of these studies are shown (Table 1). Nineteen studies were cohort studies, ten studies were retrospective case-control studies, and three studies were RCTs. The study by Tran et al. contained a nested case-control and a prospective cohort, so we analyzed the two cohorts separately. In Friedman's article, we calculated the values stratified by sex.

Nine articles were from Asia, including two from China (one from Taiwan and one from Hong Kong), five from Korea, and two from Japan. Twenty-two articles were from Western countries, including eleven from the United States, four from the United Kingdom, three from Switzerland, one from Denmark, and one from Italy. One RCT was a multicenter study from Europe, Australia, and South America. Taking into account the differences among ethnicities, we conducted a subgroup analysis in the follow-up analysis. The study population variables used for adjustment were age, sex, etiology of the potential liver disease, other complications, and the use of other drugs.

### 3.3. Quality of Included Studies

The median Newcastle–Ottawa scale score was seven (range, 5–9); eighteen of the thirty studies were considered to be of high quality. The methodological quality of all studies is described (Table 1). Most studies were adjusted for the following confounding factors: age (21/30), sex (22/30), diabetes (18/30), medications (17/30), alcoholic liver disease (16/30), viral hepatitis (13/30), and cirrhosis (12/30) (Tables 1 and 2).

### 3.4. Risk of HCC

**Figure 3:**
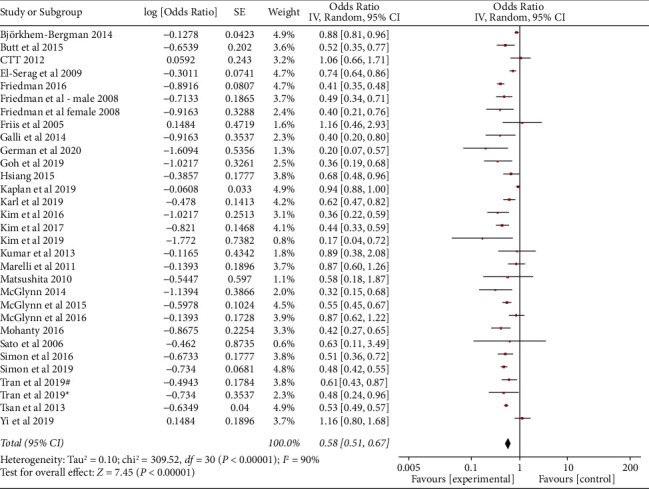
Statin and risk of hepatocellular carcinoma—adjusted OR.

**Figure 4:**
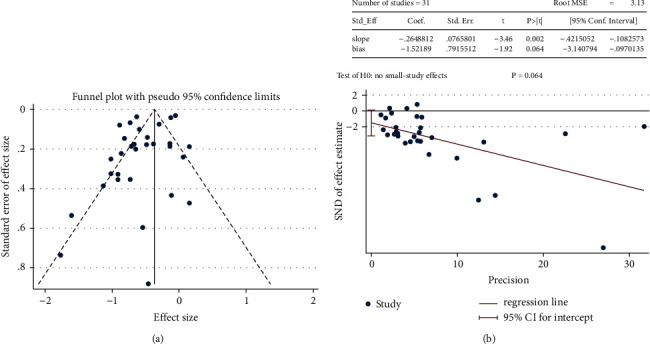
(a) Funnel plot of included studies. (b) Egger test.

In the meta-analysis assessing the risk of HCC, the use of statins was related to a significant 42% reduction in the incidence of HCC (Figure 3). There was substantial heterogeneity (Cochran's *Q* test *P* < 0.00001, *I*^2^ = 99%). The shape of the funnel plot indicated that there was a low significant publication bias (Figure 4). Results of Egger's regression test also suggested a low possibility of publication bias (*P*=0.064) (Figure 4).

### 3.5. Subgroup Analyses

Based on the predetermined assumptions, we conducted a hierarchical analysis according to the study design (Table 3). An analysis of the twenty-eight observational studies showed that the use of statins reduced the incidence of liver cancer by 43%, although the heterogeneity among the studies was large. In addition, in three studies containing data from multiple RCTs, statins did not have protective effects, although no significant heterogeneity was found in this group. This suggests that the heterogeneity of the article may be mainly concentrated on observational research.

According to the subgroup analysis stratified by geographic location, the use of statins significantly reduced the risk of HCC by 46% in Asian populations (OR, 0.54; 95% CI: 0.42–0.70) and by 40% (OR, 0.60; 95% CI: 0.51–0.71) in Western populations (although there was even greater heterogeneity). Unfortunately, these two subgroup analyses did not explain the significant heterogeneity in the overall analysis.

Next, the protective effect of statins against liver cancer caused by HBV and HCV infections was evaluated. It was confirmed that the use of statins in HBV-positive patients had a significant preventive effect against HCC (OR, 0.44, 95% CI: 0.22–0.85; *I*^2^ = 65%). Moreover, HCV-positive patients also experienced clear preventive effects (OR, 0.53, 95% CI: 0.49–0.57; *I*^2^ = 79%), and no heterogeneity was found.

After classifying the population included in the study, statins had obvious preventive effects in people with chronic liver disease (OR, 0.52, 95% CI: 0.40–0.68). However, the protective effect of statins in the general population is slightly lower than that of patients with chronic liver disease (OR, 0.60, 95% CI: 0.50–0.72).

Lipophilic statins (simvastatin, atorvastatin, fluvastatin, lovastatin, pitavastatin, and cerivastatin) were associated with a significant reduction in the incidence of HCC (OR, 0.51, 95% CI: 0.46–0.57; *I*^2^ = 23%). However, we did not find an association between hydrophilic statin drugs (pravastatin and rosuvastatin) and a reduction in the risk of liver cancer (OR, 0.77, 95% CI: 0.58–1.02; *I*^2^ = 45%). Single-drug analyses showed that simvastatin (OR, 0.53, 95% CI: 0.48–0.59; *I*^2^ = 0%), atorvastatin (OR, 0.54, 95% CI: 0.45–0.64; *I*^2^ = 0%), rosuvastatin (OR, 0.55, 95% CI: 0.37–0.83; *I*^2^ = 0%), lovastatin (OR, 0.30, 95% CI: 0.15–0.62; *I*^2^ = 0%), and pitavastatin (OR, 0.36, 95% CI: 0.17–0.75 *I*^2^ = 0%) had significant benefits, with moderate heterogeneity. However, we found no benefit of fluvastatin (OR, 0.83, 95% CI: 0.48–1.44; *I*^2^ = 10%), pravastatin (OR, 0.77, 95% CI: 0.57–1.05; *I*^2^ = 0%), or cerivastatin (OR, 0.61, 95% CI: 0.26–1.42; *I*^2^ = 0%).

Analysis of the magnitude of statin chemoprophylaxis showed a linear correlation with the dose, with AHRs of 0.55 (95% CI: 0.47–0.65) and 0.38 (95% CI: 0.28–0.50), respectively, in patients receiving cumulative defined daily doses (CDDDs) less than or greater than 365 (Table 3).

Next, we further explored the effect of coadministration of aspirin on the effect of statins on the prevention of hepatocellular carcinoma. Concomitant use of aspirin did not significantly improve the chemopreventive effect of statins (OR, 0.57; 95% CI: 0.40–0.81). In addition, aspirin alone has no chemopreventive effect on the occurrence of liver cancer (OR, 0.86; 95% CI: 0.65–1.14).

In addition, we discussed the incidence of liver cancer in the population within 10 years of follow-up and those with more than 10 years of follow-up, respectively. Interestingly, we found a lower incidence of liver cancer in the population over 10 years (OR, 0.54 vs. 0.65).

### 3.6. Sensitivity Analyses

Sensitivity analyses were conducted based on study design and quality to identify the source of the heterogeneity in observational studies (Table 4). The chemopreventive effect of statins against liver cancer was confirmed in case-control studies and cohort studies, and there was no significant difference between the groups. In the analysis based on research quality, both high-quality research and low-quality research studies have protective effects, but there is no significant difference between the two (Table 4). However, high-quality research has significant heterogeneity (*I*^2^ = 94%).

Through the analysis of research sites of high-quality observational research, it is found that Asian research has no obvious heterogeneity (*I*^2^ = 44%), while Western research has significant heterogeneity (*I*^2^ = 92%). Therefore, this explains to a certain extent that the overall heterogeneity of the article comes from high-quality Western observational research (Table 4).

## 4. Discussion

Through a comprehensive meta-analysis of all existing studies of more than 56,838 HCC patients, we found that after adjusting for confounding variables, the use of statins significantly reduces the risk of HCC by 42%. In our meta-analysis, the possibility of selection or publication bias was low, and all included articles did not meet the exclusion criteria. This meta-analysis showed that long-term use of statin can reduce the incidence of HCC, which is consistent with the previous six meta-analysis [12, 50–54].

Statins are 3-hydroxy-3-methylglutaryl CoA (HMG-CoA) reductase inhibitors that have long been used for the treatment of dyslipidemia and cardiovascular diseases. In recent years, there have been an increasing number of experimental studies on statins as potential anticancer drugs. A number of studies have shown that statins have immunomodulatory properties, antiproliferative properties that regulate cell cycle regulatory proteins, proapoptotic properties, and antiinvasive properties [4, 55–57]. Studies have shown that statins exert the antitumor effect through the dependent/independent HMG-CoA reductase pathway. Statins competitively inhibit HMG-CoA reductase, block the conversion of HMG-CoA to mevalonate, and inhibit the production of several downstream molecules, including isoprenoid. Studies have shown that statins can not only lower cholesterol but also inhibit the proliferation of cancer cells by reducing the production of isoprene (a posttranslational modification of RAS protein) [58]. Furthermore, this effect of statins was independent of its lipid-lowering effects because nonstatin lipid-lowering agents were not associated with reduction in the risk of HCC [17, 59].

In addition, chronic liver damage is also an essential factor affecting the progression of HCC [60]. Liver inflammation is mainly caused by c-Fos expressed in liver cells, and the recruited immune cells are primarily granulocytes. The main triggers of this process may be chronic damage to liver cells caused by the accumulation of oxysterols, cholesterol, and primary alkaline phosphatase. Statins can induce the secretion of IL-6, IL-1*β*, tumor necrosis factor, reactive oxygen species (ROS), and other molecules that mediate inflammatory damage, thereby further inducing hepatocyte apoptosis [61]. However, statins can inhibit the expression of TNF-*α* and IL-6 and significantly reduce ROS production and metalloproteinase activity, thereby reducing liver inflammation [62, 63]. Relevant studies have shown that the use of statins in c-Fos hep-tetOFF mice can reduce liver inflammation [64].

The activation of MYC is very important in the tumorigenesis of liver cancer, and its inactivation can lead to the continuous regression of HCC [65, 66]. In human hepatocarcinoma cell lines and aflatoxin-induced liver cancer transgenic animal models, atorvastatin has been shown to block phosphorylation and activation of Myc through HMG-CoA reductase-dependent pathways, thereby inhibiting tumorigenesis and tumor growth [57].

Statins can induce cell apoptosis, but the underlying pathway has not been fully confirmed. Statins may block the cell cycle in the G0/G1 phase by interfering with the Ras/Raf/MEK/ERK signaling pathways or regulating cyclin-dependent kinases and their inhibitors [67]. It is worth noting the results of the study by Sutter et al. showed that the apoptosis of liver cancer cells induced by statins may be related to reduction in DJM and activation of caspases 8 and 3 [68].

In addition, Raf-MEK-ERK and PI3K-AKT-mTOR pathways are necessary for cancer cell survival. Statin-induced isoprene can not only reduce the activation of these two channels but also inhibit HCV replication [69]. Therefore, the enhancement by statins of the human virological response to peginterferon and ribavirin therapy may be related to improvements in the antiviral activity of HCV polymerase and protease inhibitors [70, 71].

The chemopreventive connection between statins and liver cancer is obvious among Asians but not among Western populations, based on the data from high-quality observational studies. We can speculate that the difference may be related to the different etiologies of liver cancer in the two regions.

Eighty percent of HCC cases occur in East Asia and sub-Saharan Africa [1]. We know that the main risk factors for liver cancer in the Western population are chronic HCV infection and fatty liver disease [72]. However, in Asia, chronic HBV infection is the primary risk factor [73].

HBV participates in the occurrence and development of HCC through direct and indirect mechanisms, and the HBV protein HBx plays a major role. Compared with liver cancer caused by other risk factors, HBV-related liver cancer has a higher chromosomal mutation rate, p53 gene mutation rate, and inactivation mutation rate; fetal liver/liver progenitor cell genes are often overexpressed, and the Wnt/b-catenin pathway is often activated, but the mutation rate of activated b-catenin is low [74]. Similarly, the HCV core protein promotes cell proliferation by inhibiting E2F-1, Rb phosphorylation, p53 protein, and p21 protein kinase inhibitors [75]. The HCV core protein can also activate the Wnt/-catenin cascade to promote cell proliferation [76]. In addition, the HCVNS3 protein, which is a multifunctional protein, can bind to p53 to inhibit its activity, thereby inhibiting the transcription of p21, leading to uncontrolled cell proliferation and accelerating the occurrence and development of liver cancer [77]. Studies have found that statins can counteract these effects and inhibit tumor growth.

In Asian populations, statins mainly work by antagonizing the carcinogenic effects of HBV, while in Western populations, the mechanism is unclear. The results of our analysis also confirmed the difference in this benefit between Eastern and Western populations and the possible source of the difference. When we analyzed the subgroups of HBV-related liver cancer and HCV-related liver cancer, the results showed that statins had protective effects against both types of liver cancer, especially HBV-related liver cancer (Table 3). Increasing evidence shows that diabetes and/or insulin resistance are intrinsically linked to the progression of liver disease in patients with chronic hepatitis C, and statins may exert their antitumor effects through antiinfective mechanisms.

Further analysis showed that the chemopreventive effect of statins is more significant in patients with chronic liver disease than in the general population. Patients with chronic liver disease are at high risk of HCC. The chemopreventive effect of statins is more likely to yield positive results in people with chronic liver disease.

After further analysis of the dosage of statins, it was found that higher cumulative doses had greater chemopreventive effects than lower doses of statins (Table 3).

In a cohort study of HCV-positive patients, Tsan et al. confirmed the chemopreventive effect of statins against liver cancer [23]. Compared with patients who did not use statins, AHR for the cumulative dose over 28–89 days was 0.66, that for 90–180 days was 0.47, and that for more than 180 days was 0.33; the *P* values were all less than 0.001. Simon et al. [25] used a similar dose division method to Tsan et al. and drew the same conclusion in an HCV-positive patient cohort (AHR = 0.85 vs. 0.48 vs. 0.51). Kim et al. [26] also showed that the adjusted ORs were 0.56, 0.41, and 0.30 for patients who had cumulative defined doses of 180–365, 365–720, and greater than 720 daily doses, respectively. In their study involving a cohort of HBV-positive patients, Goh et al. confirmed that continuous high-dose statin therapy exerted a relatively stronger chemopreventive effect against liver cancer [28]. The AHRs were 0.63 (0.31–1.29), 0.51 (0.21–1.25), 0.32 (0.07–1.36), and 0.17 (0.06–0.48) for patients who had 28–365, 366–730, 731–1095, and more than 1095 cumulative daily defined doses, respectively. In the analysis of individual patient data from 5 randomized controlled trials conducted by the CTT collaborative group, compared with “less” use of statins, “more” use of statins had a statistically significant chemopreventive effect [48].

Our research showed that lipophilic statins had a chemopreventive effect against liver cancer, while hydrophilic statins did not (Table 3). As has been observed in previous individual studies, lipophilic statins (simvastatin, atorvastatin, fluvastatin, lovastatin, pitavastatin, and cerivastatin) are more effective at preventing liver cancer than hydrophilic statins (49% vs. 23%). It can be speculated that the reason lipophilic statins (such as lovastatin and simvastatin) have stronger chemoprotective effects is that they have better fat solubility and membrane permeability than hydrophilic statins (such as pravastatin). This theory is supported by our results to a certain extent. The study of Li et al. [78] also showed that the use of lipophilic statins is associated with a lower risk of HCC in patients with HBV or HCV infection, but has nothing to do with hydrophilic statins, which coincides with our results. Further analysis of different types of statins found that lovastatin has the best protective effect, while fluvastatin has no protective effect (Table 3). This conclusion is different from the study of Zhou et al. [51]. Their study shows that fluvastatin has the best protective effect. Considering that their study only included 5 articles, our conclusion is more reliable because the conclusion is more stable. In addition, the study by Facciorusso et al. showed that both simvastatin (OR, 0.69; 95% CI: 0.42–1.15) and rosuvastatin (OR, 0.53; 95% CI: 0.04–6.38) had no chemopreventive effect, which is contrary to our conclusion [54]. Our study shows that both simvastatin (OR, 0.53; 95% CI: 0.48–0.59) and rosuvastatin (OR, 0.55; 95% CI: 0.37–0.83) have chemopreventive effects. Considering that the study by Facciorusso et al. included only two studies for both types of statin analysis, the lack of representativeness due to the small number of included studies may cause errors.

Our study showed that coadministration of aspirin did not significantly improve the preventive effect of statins on liver cancer (OR, 0.57 vs. 0.58), and aspirin alone had no preventive effect on liver cancer (OR, 0.86; 95% CI: 0.65–1.14). This conclusion contradicts previous studies. Studies by Luca et al. have shown that low-dose aspirin has a preventive effect on a variety of malignancies, including hepatocellular carcinoma [79]. The reason for this contradiction may be that our study included fewer studies, thus making the conclusions less reliable and representative.

Interestingly, we analyzed the relationship between follow-up time and HCC incidence, and the results were different from our assumptions. According to the general assumption, with the increase of follow-up time, the incidence of liver cancer will also increase. However, our analysis showed that the incidence of liver cancer was lower in the population with a follow-up period of more than 10 years than in the population with a follow-up period of fewer than 10 years (OR, 0.54 vs. 0.65). Such results demonstrate the effect of statins in preventing liver cancer from another aspect because with the increase of follow-up time, the time of taking statins will theoretically increase, thus improving its prevention effect of liver cancer, and the incidence of liver cancer will decrease accordingly.

Alcohol is a recognized carcinogen and is closely related to the occurrence of liver cancer [80]. Therefore, we also attempted to further analyze the potential impact of alcohol intake on the protective effect of statins on hepatocarcinogenesis. Of the 28 observational studies included, 13 included alcohol intake in the population, 8 of which were cohort studies. Data on alcohol intake were presented in aggregated demographic characteristics, so we were unable to extract specific values for alcohol intake and the risk of statins and liver cancer, so we could not perform a meta-analysis of their potential risk. However, a previous study by Wei-Che et al. showed that statin use may reduce the risk of HCC in alcohol abusers [81]. Therefore, we also have reasons to infer that statin is a protective factor for liver cancer in alcohol abusers and hope that more high-quality RCT studies can be carried out in the future to facilitate the exploration of this issue.

This analysis mainly included observational studies. The number of people included in observational studies accounted for 96.99% of the total number of patients, and a significant chemopreventive effect was observed in observational studies (OR, 0.57, 95% CI: 0.49–0.66). RCTs accounted for a relatively small proportion of the included studies, and no obvious chemopreventive effect was observed (OR, 0.95, 95% CI: 0.62–1.45).

We should consider the results of the RCTs included in the context. Most of the RCTs involved Western population, and the different etiologies of liver cancer may have weakened the chemopreventive effect of statins. Moreover, long-term RCTs with large populations are difficult to conduct, and there was a risk of loss to follow-up. This should also be considered.

In addition, the results of this study are significantly different from those of Yi et al. [27]. Research by Yi et al. showed that the lower risk of hepatocellular carcinoma is related to higher cholesterol levels and not the use of statins. In their study, after adjusting cholesterol, the protective effect of statins disappeared (HR = 1.16, 95% CI = 0.80–1.69). Since cholesterol levels are significantly negatively correlated with the occurrence of liver cancer, the adjustment of cholesterol before and after the study is also very important.

### 4.1. Strength and Limitations

This study included more than 4.9 million participants, which is the largest study we have known so far. Compared with the previous meta-analysis, we focused on the relationship between the use of statins and the risk of liver cancer. Due to the inclusion of a large number of studies, the stability and persuasiveness of the results have increased. In addition, this study conducted a comprehensive discussion on potential confounding factors and demonstrated in detail the study design, study area, research quality, virus type, population characteristics, and cumulative defined daily dose. These arguments explain to a certain extent that the overall heterogeneity of the article comes from high-quality Western observational research and provide an improved direction for subsequent research design.

In addition to the limitations formerly mentioned, other limitations deserve further consideration. First, the incidence of cardiovascular events in people with chronic liver disease is correspondingly higher [82], so they may choose to use more statins, which will cause deviation. Second, although the publication bias of this article is low, it is still worthy of further exploration because negative research related to liver cancer by statins may be more difficult to publish. Third, the studies included were mostly observational (cohort studies and case-control studies), and there were few RCTs. The main reason is that it is very difficult to implement large-scale RCTs. In addition, the RCTs included in this analysis were mainly from Western countries, which may have affected the results. Finally, differences in language, lifestyle, social class, education level, and sample size among studies may also have resulted in bias. Fourth, in the subgroup analysis of different types of statins, due to insufficient relevant research, the number of literature included in each group is relatively small. Therefore, it is necessary to carry out relevant research on different types of statins in the future, which will have a positive significance for clinical treatment.

## 5. Conclusion

Overall, this meta-analysis showed that the chemopreventive effect of statins on liver cancer is more pronounced in Asian populations than in other populations. Due to the differences in the etiology of liver cancer between Eastern and Western populations, we should carefully judge the benefits of statins in Western populations. At present, the relevant large-scale RCTs have mainly been performed in Western countries. In the future, it is necessary to carry out RCTs in areas with high burdens of liver cancer (such as East Asia). The chemopreventive effects of statins require their long-term use, and the burden imposed by the cumulative cost may have a certain passive influence on their clinical use. However, in areas with high burdens of liver cancer, the preventive use of statins should be considered.

## Figures and Tables

**Table 1:** Characteristics and quality of included studies assessing the risk of HCC with statin use.

Study	Design	Location	Setting	Time period	Total no. of subjects	No. of HCC cases	Variables adjusted for^a^	Study quality^b^		
								Selection	Comparability	Outcome/exposure
*Observational studies*										
Karl et al. (2019)	Cohort	Swedish	Population based	1998–2012	2,440,620	2742	1, 2, 7, 9, 12, 13, 18, 21	^*∗∗∗*^	^*∗∗*^	^*∗*^
Friis et al. (2005)	Cohort	Danish	Population based	1989–2002	348262	171	1, 2, 9, 25	^*∗∗*^	^*∗∗*^	^*∗∗*^
Tsan et al. (2013)	Cohort	Taiwan	Population based	1999–2010	260864	27883	1, 2, 5, 7, 11	^*∗∗∗*^	^*∗∗*^	^*∗∗∗*^
Butt et al. (2015)	Cohort	United States	Population based	2002–2013	7248	141	16	^*∗∗*^	^*∗∗*^	^*∗∗*^
Simon et al. (2016)	Cohort	United States	Population based	2001–2014	9,135	233	1, 2, 4, 6, 7, 8, 9,10, 12, 13, 16, 22, 26	^*∗∗∗*^	^*∗∗*^	^*∗∗*^
Kim et al. (2017)	Case-control	Korea	Population based	2002–2013	9,852	1642	5, 6, 7, 9, 11, 12, 13, 20	^*∗∗*^	^*∗∗*^	^*∗∗∗*^
Yi et al. (2019)	Cohort	Korea	Population based	2004–2007	400,318	1686	1, 2, 3,4, 5, 6, 7, 12, 13, 21, 28	^*∗∗∗*^	^*∗*^	^*∗∗*^
Goh et al. (2019)	Cohort	Korea	Population based	2008–2012	7,713	702	1, 2, 3, 5, 7, 9, 18, 21, 22	^*∗∗*^	^*∗∗*^	^*∗∗∗*^
El-Serag et al. (2009)	Case-control	United States	Population based	1997–2002	6,515	1303	3, 4, 5, 6, 8, 9, 10	^*∗∗∗*^	^*∗∗*^	^*∗*^
McGlynn et al. (2015)	Case-control	UK	Population based	1988–2011	5,835	1195	3, 4, 6, 7, 9, 12, 13, 31	^*∗∗∗∗*^	^*∗*^	^*∗*^
Tran et al. (2019)^#^	Case-control	UK	Population based	2000–2011	2,537	434	1, 2, 6, 9, 13, 14, 23	^*∗∗∗*^	^*∗∗*^	^*∗∗*^
Tran et al. (2019)^*∗*^	Cohort	UK	Population based	2006–2010	471,851	182	1, 2, 6, 9, 13, 14, 23	^*∗∗∗*^	^*∗∗*^	^*∗∗*^
Marelli et al. (2011)	Cohort	United States	Population based	1990–2009	91,714	105	1, 2, 8, 12, 13, 14	^*∗∗∗∗*^	^*∗∗*^	^*∗∗∗*^
Friedman et al. (2008)	Cohort	United States	Population based	1994–2003	361,859	42	14	^*∗∗∗∗*^	/	^*∗∗*^
Hsiang (2015)	Cohort	Hong Kong	Population based	2000–2012	53,513	6883	1, 2, 5, 7, 14	^*∗∗∗∗*^	^*∗∗*^	^*∗∗*^
Mohanty (2016)	Cohort	United States	Population based	1996–2009	40,512	173	1, 2,7, 8, 12, 19, 24	^*∗∗∗*^	^*∗∗*^	^*∗∗*^
Björkhem-Bergman (2014)	Case-control	Swedish	Population based	2006–2010	23,964	3994	1, 2, 3, 4, 6, 7, 9, 21, 17	^*∗∗∗*^	^*∗∗*^	^*∗∗*^
Friedman (2016)	Case-control	United States	Population based	1996–2014	145,727	2,877	3, 4, 6, 9, 12, 14, 17	^*∗∗∗*^	^*∗∗*^	^*∗*^
McGlynn (2014)	Case-control	United States	Population based	1999–2010	562	94	4, 6, 8, 14, 17	^*∗∗*^	^*∗∗*^	^*∗∗∗*^
German et al. (2020)	Case-control	United States	Population based	2002–2016	102	34	1, 2, 9	^*∗∗*^	^*∗∗*^	^*∗*^
Galli et al. (2014)	Cohort	Italy	Population based	1991–2012	5357	19	NR	^*∗∗*^	^*∗∗*^	^*∗∗*^
Kumar et al. (2013)	Cohort	United States	Population based	1988–2011	243	29	7, 14, 17, 24	^*∗∗*^	^*∗∗*^	^*∗∗*^
McGlynn et al. (2016)	Case-control	UK	Population based	1988–2011	1657	339	3, 4, 6, 7, 12, 13, 31	^*∗∗∗*^	^*∗∗*^	^*∗∗*^
Kim et al. (2016)	Case-control	Korea	Population based	2002–2013	1374	229	3, 4, 5, 6, 9, 11, 20, 34, 35	^*∗∗∗*^	^*∗∗*^	^*∗∗*^
Kim et al. (2019)	Cohort	Korea	Population based	2002–2003	13063	193	1, 2, 6, 12, 13, 18, 32, 33	^*∗∗*^	^*∗∗*^	^*∗∗*^
Kaplan et al. (2019)	Cohort	United States	Population based	2008–2016	74,984	2420	3, 4, 6, 7, 8, 12, 13, 18, 20, 32, 36, 37	^*∗∗∗∗*^	^*∗∗*^	^*∗∗∗*^
Simon et al. (2019)	Cohort	Swedish	Population based	2005–2013	16 668	1012	NR	^*∗∗∗∗*^	^*∗∗*^	^*∗∗∗*^
*RCTs*										
Matsushita et al. (2010)	RCT	Japan	Individual patient data analysis of trials	2010	13,724	12	NR	^*∗∗∗∗*^	^*∗∗*^	^*∗*^
CTT (2012)	RCT	Europe, Australia, North America	Individual patient data analysis of trials	2012	134,537	68	NR	^*∗∗∗∗*^	^*∗∗*^	^*∗∗*^
Sato et al. (2006)	RCT	Japan	Secondary analysis of RCT	1991–1995	263	1	1, 2, 13	^*∗∗*^	^*∗∗*^	^*∗*^

N/A, not applicable. ^a^1, age; 2, sex; 3, HBV; 4, HCV; 5, cirrhosis; 6, alcoholic liver disease/alcohol use; 7, diabetes mellitus; 8, race; 9, other medications (aspirin/nonsteroidal anti-inflammatory medications, angiotensin-converting enzyme inhibitors, metformin, antidiabetic medications, PPIs, H2RAs, antihypertension medications, paracetamol, insulin, thiazolidinedione, and sulfonylurea); 10, other lipid-lowering agents; 11, socioeconomic status; 12, body mass index; 13, smoking; 14, comorbidities; 15, calendar year; 16, FIB-4 score; 17, other liver disease etiology; 18, hypertension; 19, dyslipidemia/hyperlipidemia/hypercholesterolemia; 20, CCI index; 21, complete biochemical tests; 21, education level; 22, antiviral therapy/attainment of SVR; 23, obesity; 24, MELD score; 25, hormone replacement therapy; 26, caffeine intake; 27, the presence of nonhemorrhagic varices; 28, physical activity; 29, follow-up duration; 30, gout; 31, rare metabolic disorders; 32, biochemical indicators; 33, family history of liver disease; 34, previous cancer; 35, pulmonary disease; 36, history of substance abuse; 37, center characteristics. ^b^Study quality assessment of observational studies was performed using the Newcastle–Ottawa scale; each asterisk represents if an individual criterion within the subsection was fulfilled.

**Table 2:** Comparison of reported baseline risk factors for HCC and analysis of potential confounders in included studies.

Study	Age (*y*)		Sex (% male)		Diabetes (% total)		Cirrhosis (% total)		HBV/HCV (% total)		Alcoholic liver disease or alcohol use (% total)		Angiotensin-converting enzyme inhibitor/nonsteroidal anti-inflammatory drug/aspirin (% total)		Nonstatin lipid-lowering drug (% total)	
	Case	Control	Case	Control	Case	Control	Case	Control	Case	Control	Case	Control	Case	Control	Case	Control
Karl et al. (2019)	NR		NR		NR		NR		NR		NR		NR		NR	
Friis et al. (2005)	60.7	46.6	57	50	NR		NR		NR		NR		NR/NR/80	NR/NR/48	NR	
Tsan et al. (2013)	53.9	49.8	49.8	50.2	56.5	23.1	9.6	19.7	0/100	0/100	8.8	11.5	53.9/81.4/58.9	25.1/67.0/24.8	6.6	0.5
Butt et al. (2015)	53	52	96.4	94.9	22.1	6.8	17.3	25.2	0/100	0/100	30.4	33.2	NR		NR	
Simon et al. (2016)	53.5	52.5	96.16	95.37	24.03	8.87	14.02	21.43	0/100	0/100	34.81	38.79	65.7/NR/NR	38.11/NR/NR	15.53	4.02
Kim et al. (2017)	61.8	61.8	83.6	83.6	18.6	11.2	34.2	1.1	NR		16.9	5.1	NR/NR/17.7	NR/NR/20.4	NR	
Yi et al. (2019)	NR		NR		NR		NR		NR		NR		NR		NR	
Goh et al. (2019)	50	47	67.6	66.1	39.6	10.9	14	25.1	100/0	100/0	NR		NR		NR	
El-Serag et al. (2009)	72	72	99	99	100	100	28.2^a^	1.6	1.9/14.7	0.2/1.8	16.5	1.2	64/21.2/44.6	67.4/20.6/47.9	4.1	3.9
McGlynn et al. (2015)	67.2	67	71.6	71.6	29	10	NR		6.2	0.1	15.8	4	NR		NR	
Tran et al. (2019)^#^	NR		67.3	67.1	12.2	4.2	NR		NR		55.7	56.9	NR/36.0/36.4	NR/36.1/31.3	NR	
Tran et al. (2019)^*∗*^	NR		62.6	46.1	19.2	5	NR		NR		83.5	91.7	NR/12.6/23.6	NR/16.3/13.7	NR	
Marelli et al. (2011)	64.2	64.2	52.2	52.6	16.1	15.8	NR		0.06	0.06	NR		—/28.4/19.4	—/28.2/19.6	NR	
Friedman et al. (2008)^a^	NR		NR		NR		NR		NR		NR		NR		NR	
Hsiang (2015)	58.7	37.6	67.9	25.5	45.1	7.4	2.7	1.6	NR		NR		53.1/20.6/NR	3.5/1.8/NR	NR	
Mohanty (2016)	56	54	98.3	97.7	54.8	28.9	NR		NR		52.5	56.6	NR		NR	
Björkhem-Bergman (2014)	NR		52	52	NR		NR		NR		NR		NR		NR	
Friedman (2016)	NR		NR		NR		NR		NR		NR		NR		NR	
McGlynn (2014)			74.47	74.36	42.55	25.85	NR		1.06/48.94	0.21/1.71	25.53	0.85	NR		NR	
German et al. (2020)	64.3	65.2	64.7	64.7	64.7	76.5	91	98	NR		NR		26.5/NR/41.2	48.5/NR/58.8	NR	
Yang et al. (2013)	NR		NR		NR		NR		NR		NR		NR		NR	
Galli et al. (2014)	51.1	45.7	80	76	NR		NR		6/14	6/32	NR		NR		NR	
Kumar et al. (2013)	59.79	59.64	54.32	54.32	55.56	30.86	100	100	2.47/22.22	6.17/33.95	22.22	24.07	NR		NR	
McGlynn et al. (2016)	68.1	67.9	NR		17.1	7.89	NR		2.1	0.1	4.4	1.4	NR/NR/28.3	NR/NR/24.1	NR	
Kim et al. (2016)	NR		81.4	81.4	100	100	40.6	2.5	NR		11.8	8.7	NR/NR/23.6	NR/NR/29.3	NR	
Kim et al. (2019)	55.4	51.8	41.1	51.8	NR		0	0	100/0	100/0	31.2	38	NR		NR	
Kaplan et al. (2019)	64	NR	97.5	NR	70.8	40.6	53.2	34.2	NR/11.2	NR/19	38.6	29.2	NR		NR	
Simon et al. (2019)	NR		65.6	34.8/32.8	30.5	30.6/30.0	10.7	10.7/10.8	11.0/13.7	NR	14.1	14.0/14.3	NR/NR/35.5	NR/NR/35.4/36.0	NR	
Matsushita et al. (2010)	57.9	57.1	52.6	50.5	19.7	21.5	NR		NR		NR		NR		NR	
CTT (2012)	63		71		NR		NR		NR		NR		NR		NR	
Sato et al. (2006)	NR		81.7		NR		NR		NR		NR		NR		NR	

Note: for case-control study design, case refers to patients with HCC and control refers to patients without HCC; for cohort study design, case refers to statin users and control refers to statin nonusers. NR, not reported. ^#^Data source: the primary care clinical informatics unit (PCCIU) database. ^*∗*^Data source: the UK Biobank. ^a^Separate analyses of male and female subjects.

**Table 3:** Subgroup analysis to examine sources of heterogeneity observed in summary estimate.

Subgroup analysis		No. of studies	Adjusted OR	95% CI	Tests of heterogeneity			Heterogeneity between groups (*P*)
					*P*		*I* ^2^ (%)	
Study design								
Observational		28	0.57	0.49–0.66	<0.00001		91	0.03^a^
RCT		3	0.95	0.62–1.45	0.57		0	
Study location								
Asian		9	0.54	0.42–0.70	0.0008		70	0.48
Western		22	0.60	0.51–0.71	<0.00001		90	
Etiology of liver disease								
HBV		3	0.44	0.22–0.85	0.06		65	0.58
HCV		4	0.53	0.49–0.57	0.79		0	
Chronic liver disease								
Yes		11	0.52	0.40–0.68	<0.00001		95	0.42
No		18	0.60	0.50–0.72	<0.00001		86	
Molecule								
Lipophilic	7		0.51	0.46–0.57		0.16	23	0.007^a^
Hydrophilic	6		0.77	0.58–1.02		0.06	45	
Simvastatin	6		0.53	0.48–0.59		0.77	0	0.12
Atorvastatin	5		0.54	0.45–0.64		0.56	0	
Fluvastatin	3		0.83	0.48–1.44		0.33	10	
Pravastatin	5		0.77	0.57–1.05		0.71	0	
Rosuvastatin	4		0.55	0.37–0.83		0.55	0	
Lovastatin	2		0.30	0.15–0.62		0.36	0	
Pitavastatin	2		0.36	0.17–0.75		0.54	0	
Cerivastatin	2		0.61	0.26–1.42		0.34	0	
Cumulative defined daily dose								
≤365	6		0.55	0.47–0.65		0.04	47	0.02^a^
>365	4		0.38	0.28–0.50		0.44	0	
Statin combined with aspirin								
Statin and aspirin	2		0.57	0.40–0.81		<0.00001	92	0.08^a^
Just aspirin	4		0.86	0.65–1.14		0.02	69	
Time period								
≤10 years	10		0.65	0.52–0.80		<0.00001	92	0.16
>10 years	18		0.54	0.48–0.61		0.0003	62	

^a^ *P* ≤ 0.10, explains source of heterogeneity between groups.

**Table 4:** Sensitivity analysis to examine sources of heterogeneity observed in summary estimate.

Sensitivity analysis	No. of studies	Adjusted OR	95% CI	Tests of heterogeneity		Heterogeneity between groups (*P*)
				*P*	*I* ^2^ (%)	
Sensitivity analysis (to examine source of heterogeneity seen in observational studies)						
Study quality						
High quality	15	0.58	0.48–0.70	<0.00001	94	0.86
Low quality	13	0.56	0.45–0.70	<0.00001	79	
Study design						
Cohort	18	0.59	0.48–0.72	<0.00001	91	0.63
Case-control	10	0.54	0.42–0.70	<0.00001	92	
Sensitivity analysis (to examine source of heterogeneity seen in high-quality observational studies)						
Study location						
Asian	5	0.50	0.42–0.59	0.13	44	0.07^a^
Western	10	0.64	0.52–0.79	<0.00001	92	

^a^ *P* ≤ 0.10, source of heterogeneity between groups.

## Data Availability

The data used to support the findings of this study are included within the article.

## Abbreviations

BMI: Body mass index
CDDDs: Cumulative defined daily doses
DM: Diabetes
HBV: Hepatitis B virus
HCC: Hepatocellular carcinoma
HCV: Hepatitis C virus
HR: Hazard ratio
HMG-CoA: 3-Hydroxy-3-methylglutaryl CoA
MOOSE: Meta-analysis Of Observational Studies in Epidemiology
OR: Odds ratio
PPIs: Proton pump inhibitors
PRISMA: Preferred Reporting Items for Systematic Review and Meta-Analysis
RCT: Randomized controlled trials
ROS: Reactive oxygen species.
